# Dexmedetomidine administration is associated with improved outcomes in critically ill patients with acute myocardial infarction partly through its anti-inflammatory activity

**DOI:** 10.3389/fphar.2024.1428210

**Published:** 2024-08-22

**Authors:** Yimou Liu, Qian Chen, Tianyang Hu, Changming Deng, Jing Huang

**Affiliations:** ^1^ Department of Cardiology, The Second Affiliated Hospital of Chongqing Medical University, Chongqing, China; ^2^ Department of Oncology, The Second Affiliated Hospital of Chongqing Medical University, Chongqing, China; ^3^ Precision Medicine Center, The Second Affiliated Hospital of Chongqing Medical University, Chongqing, China

**Keywords:** dexmedetomidine, acute myocardial infarction, mortality, inflammation, causal mediation analysis

## Abstract

**Background:**

Dexmedetomidine (DEX) is a commonly used sedative in the intensive care unit and has demonstrated cardioprotective properties against ischemia-reperfusion injury in preclinical studies. However, the protective effects of early treatment of DEX in patients with acute myocardial infarction (AMI) and its underlying mechanism are still not fully understood. This study aims to investigate the association between early DEX treatment and in-hospital mortality in patients with AMI, and to explore the potential mediating role of white blood cell (WBC) reduction in this relationship.

**Methods:**

A retrospective cohort analysis was conducted using the Medical Information Mart for Intensive Care IV (MIMIC-IV) database. Patients with AMI were divided into the DEX and non-DEX group, based on whether they received DEX treatment in the early stage of hospitalization. The primary outcome measured was in-hospital mortality. The study evaluated the association between DEX use and in-hospital mortality using the Kaplan-Meier (KM) method and Cox proportional hazards model. Additionally, 1:1 propensity score matching (PSM) was conducted to validate the results. Furthermore, causal mediation analysis (CMA) was utilized to explore potential causal pathways mediated by WBC reduction between early DEX use and the primary outcome.

**Results:**

This study analyzed data from 2,781 patients, with 355 in the DEX group and 2,426 in the non-DEX group. KM survival analysis revealed a significantly lower in-hospital mortality rate in the DEX group compared to the non-DEX group. After adjusting for multiple confounding factors, the Cox regression model demonstrated a significant positive impact of DEX on the risk of in-hospital mortality in patients with AMI, with hazard ratios (HR) of 0.50 (95% confidence interval (CI): 0.35–0.71, *p* < 0.0001). PSM analysis confirmed these results, showing HR of 0.49 (95% CI: 0.31–0.77, *p* = 0.0022). Additionally, CMA indicated that 13.7% (95% CI: 1.8%–46.9%, *p* = 0.022) of the beneficial effect of DEX on reducing in-hospital mortality in patients with AMI was mediated by the reduction in WBC.

**Conclusion:**

The treatment of DEX was associated with a lower risk of in-hospital mortality in patients with AMI, potentially due to its anti-inflammatory properties.

## 1 Introduction

Acute myocardial infarction (AMI) is a form of myocardial necrosis resulting from acute coronary artery occlusion and is a significant public health concern globally, endangering the physical and mental health of more than seven million people annually (Thygesen et al., 2012; Reed et al., 2017). While advancements in coronary revascularization and evidence-based therapies have led to improved clinical outcomes for patients with myocardial infarction in recent decades, the overall prognosis for AMI patients, particularly those requiring intensive care, remains suboptimal (Carroll et al., 2016; Parhar et al., 2018). Research findings have indicated that the in-hospital mortality rate for patients with AMI admitted to the intensive care unit (ICU) can reach up to 25.6% (Ohbe et al., 2022). With this in mind, there is a pressing need to identify effective interventions to reduce mortality in critically ill patients with AMI.

The initial ischemic injury to the heart triggers a robust inflammatory response, which is a significant factor contributing to cardiomyocyte damage (Frangogiannis et al., 2002; Frangogiannis, 2008; Frangogiannis, 2012). Studies in large animal models have shown that the early infiltration of leukocytes into the infarcted myocardium can lead to cytotoxic damage to viable cardiomyocytes, thus prolonging ischemic injury (Entman et al., 1991). Clinical research over the past few decades has consistently demonstrated a strong link between inflammatory markers and negative outcomes in patients with AMI (Kosuge et al., 2004; Palmerini et al., 2011; Chen et al., 2023). These findings have inspired numerous clinical trials focused on enhancing outcomes in patients with AMI by early suppression of key inflammatory signals. However, clinical studies of methylprednisolone in the treatment of AMI patients have shown disappointing outcomes (Roberts et al., 1976). Subsequent trials of anti-CD18 integrin approaches (Faxon et al., 2002) and complement inhibition strategies (Armstrong et al., 2007) have also yielded unsatisfactory results, indicating the need for more targeted and efficient anti-inflammatory interventions.

Dexmedetomidine (DEX) is a highly selective α2 adrenergic receptor agonist known for its sedative, analgesic, anti-anxiety, and anti-inflammatory properties, making it a common choice in perioperative and intensive care settings (Keating, 2015; Bilotta and Pugliese, 2020; Homberg et al., 2023). Recent research has highlighted DEX as a cardioprotective agent against ischemia-reperfusion injury (IRI) (Takahashi et al., 2023). One of the many ways in which DEX exerts cardioprotective effects is by reducing myocardial inflammation. Studies have shown that DEX can downregulate the expression of high mobility group box 1-toll-like receptor 4-nuclear factor κB, decrease levels of pro-inflammatory factors like TNF-α and IL-6, and enhance anti-inflammatory effects (Yang et al., 2017). Many clinical trials to date have shown promising results in reducing myocardial damage following cardiac surgery and improving patient prognoses (Ji et al., 2013; Peng et al., 2021; Chen et al., 2022), although a few conflicting outcomes have been reported in some studies (Tosun et al., 2013; Kim et al., 2014). Notably, there is a lack of clinical data on DEX’s impact on patients with AMI, warranting further investigation.

This study aimed to explore the potential benefits of using DEX in patients with AMI during their time in the ICU. Through causal mediation analysis (CMA), we also examined whether the cardioprotective properties of DEX were linked to its anti-inflammatory effects.

## 2 Materials and methods

### 2.1 Data source

Data for this study were obtained from the Medical Information Mart for Intensive Care IV (MIMIC-IV), an updated version of the MIMIC-III that was released on 6 January 2023 (https://mimic-iv.mit.edu/). MIMIC-IV is clinical critical care database that makes the records of over 50,000 patients at Boston, Massachusetts’ Beth Israel Deaconess Medical Center from 2008 to 2019 available online. Patient privacy was safeguarded through the use of anonymous personal identifiers, eliminating the need for informed consent. Access to the database was granted to the author upon completion of relevant courses and receipt of the necessary certification (no. 61895238).

### 2.2 Participants

This was a large retrospective cohort study. All included cases were diagnosed with AMI using International Classification of Diseases 9 and 10 codes. All participants were 18 years of age or older. Patients with a hospital or ICU stay of less than 48 h, and those with more than 20% missing information, were excluded from the analysis. To mitigate confounding factors related to prolonged ICU stays, the study specifically focused on patients in whom DEX was initiated soon after ICU admission and excluded those who started the medication 48 h or more after admission. Eligible patients were categorized into two groups: those who received DEX within 48 h of ICU admission and those who did not receive the medication during their ICU treatment (non-DEX).

### 2.3 Data extraction

After determining the stay identity of the selected patients, data extraction was performed using Structured Query Language (SQL). The SQL script code was obtained from the GitHub website (https://github.com/MIT-LCP/mimic-iv). Clinical variables included study participants’ demographic information, vital signs, laboratory parameters, type of myocardial infarction, and comorbidities. We extracted the first measurement parameters from data gathered within 24 h of admission to the ICU. Additionally, clinical scores such as the Sequential Organ Failure Assessment score (Vincent et al., 1998), Simplified Acute Physiology Score II (Le Gall et al., 1993), and Richmond Agitation-Sedation Scale score (Sessler et al., 2002) were included in the analysis. Clinical treatment information was also collected, encompassing drug treatment, revascularization therapy, renal replacement therapy and mechanical ventilation.

Detailed information on DEX and vasoactive drugs was also gathered, including drug name, dose, route, and start and end times. We used the vasoactive-inotropic score (Gaies et al., 2010) to standardize the various vasoactive medications administered to patients and assess the level of circulatory support provided.

### 2.4 Outcomes

The primary outcome of this study was the measurement of all-cause in-hospital mortality, with secondary outcome data including 30- and 90-day all-cause mortality, the patients’ lengths of ICU stays, lengths of hospital stays, and the incidence of acute kidney injury (AKI) within 7 days of admission. We were also somewhat concerned with adverse events related to DEX, specifically bradycardia and hypotension.

### 2.5 Statistical analysis

Normality tests indicated that all continuous variables in this study did not follow a normal distribution; therefore, they are presented as medians and quartiles. Comparisons between groups were performed using the χ^2^ test or Fisher’s exact test for categorical variables and the Mann-Whitney U test for continuous variables.

To determine the impact of DEX treatment on survival outcomes, Kaplan–Meier curves and the log-rank test were used. Cox regression models were used to assess the impact of DEX treatment on survival prognosis, controlling for various confounding factors. The hazard ratio (HR) and 95% confidence interval were calculated to provide a comprehensive evaluation. Before the multivariate Cox regression was noted, its proportional hazards assumption was assessed using the Schoenfeld residual method. Furthermore, potential multicollinearity among the independent variables was investigated by calculating the variance inflation factors (Gaies et al., 2010). Logistic regression models were used to assess the impact of DEX on the incidence of AKI, hypotension, and bradycardia throughout the ICU stay. Linear regression was employed to examine the correlation between DEX use and the lengths of ICU and hospital stays. In the multivariate regression analysis, baseline variables that were considered clinically relevant or univariately related to prognosis (*p* < 0.05) were selected as adjusted covariates. Stepwise regression was used to present the results for the primary outcome.

This study used propensity score matching (Zhang, 2017) to adjust covariates and ensure the robustness of the findings. The DEX and non-DEX groups were matched using propensity score-based 1:1 nearest neighbor matching with a caliper width of 0.2 SDs. Standardized mean differences (SMDs) were calculated to assess the covariate balance before and after matching, with an SMD greater than 0.1 indicating a relevant imbalance. Cox regression was then applied to the matched cohorts.

To investigate whether the impact of DEX on the primary outcome was mediated by its anti-inflammatory properties, we conducted a CMA (Imai et al., 2010), using changes in white blood cell counts as the mediating variable (defined as the white blood cell count on Day 4 minus admission). The CMA separated the total effect of DEX medication on in-hospital mortality into direct effects and indirect effects, expressed as average direct effects (ADE) and average causally mediated effects (ACME), respectively. ADE represented the direct association between DEX use and the primary outcome, independent of the mediator, keeping the mediated pathway fixed. ACME signified the indirect relationship between DEX use and outcomes by the mediator. This approach provided more comprehensive insights, compared to traditional correlation analyses.

In addition, subgroup analyses were conducted considering variables such as age, gender, race, type of myocardial infarction, revascularization and comorbidities. To analyze the dose-response relationship, the study also examined the relationship between the average dose rates of DEX and the cumulative dose over a 7-day period in relation to in-hospital mortality.

Missing values in the data were imputed using the iterative interpolation method based on random forest, with the R missForest package (Stekhoven and Bühlmann, 2012). Multiple imputed data sets were created with different interpolated values for statistical analysis, and the final results were combined to provide valid estimates.

Statistical significance was defined as a two-sided probability value *p* < 0.05. All analyses were performed using R software (version 4.1.0) or Stata (version 14.0).

## 3 Results

### 3.1 Baseline characteristics

**FIGURE 1:**
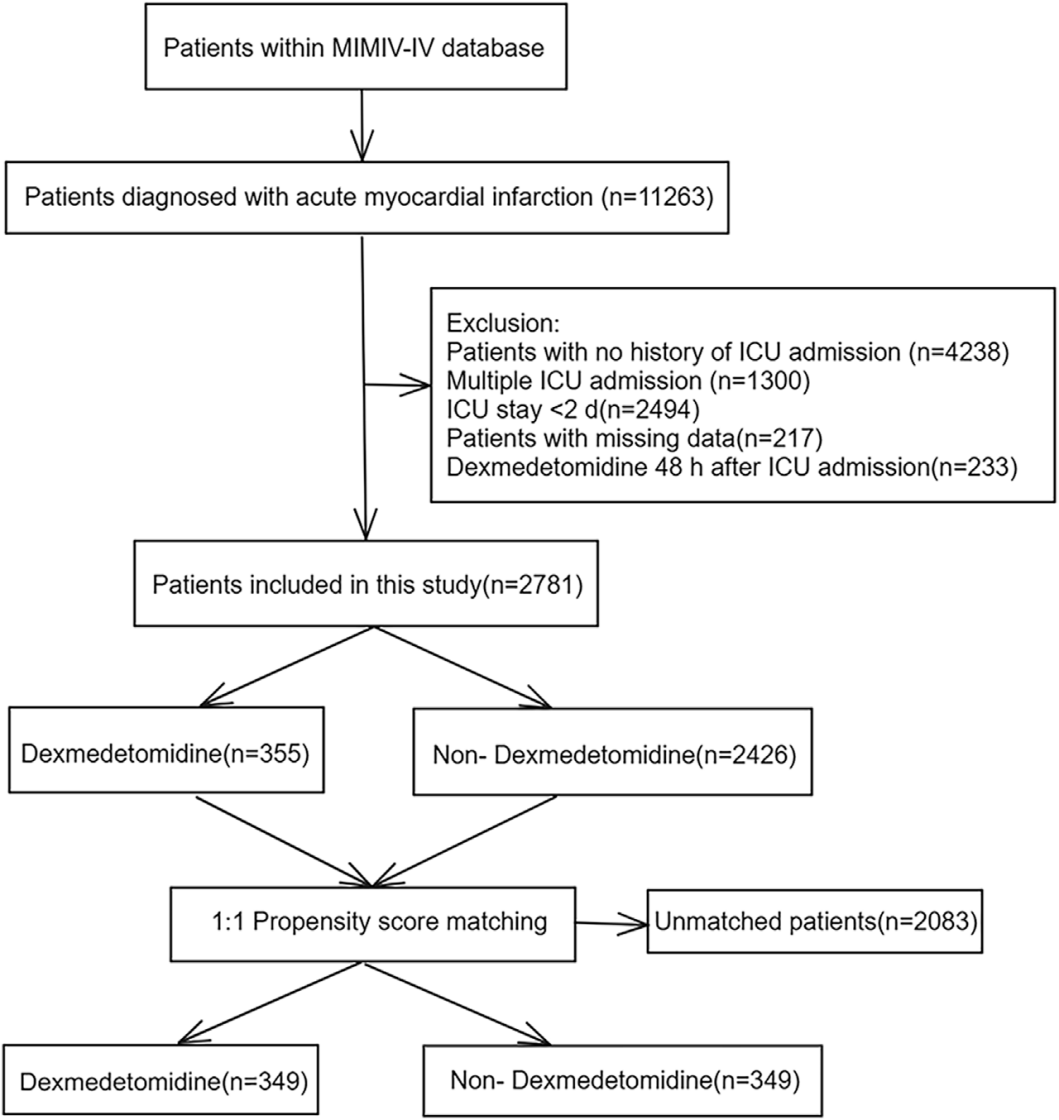
Flowchart of the study. MIMIC IV: Medical Information Mart for Intensive Care IV; ICU: intensive care unit.

A total of 11,263 hospitalization data of patients with MI were reviewed, with 8,482 records excluded based on the exclusion criteria (Figure 1). The study ultimately included 2,781 patients, among whom 355 (12.8%) received DEX during hospitalization. Table 1 displays the baseline characteristics of the DEX group compared to the non-DEX group in the original cohort.

**TABLE 1:** Baseline characteristics between two groups before PSM.

Before PSM				
Characteristics	Non-DEX group	DEX group	*p*-value	SMD
N	(n = 2,426)	(n = 355)		
Age	73.8 (64.6, 82.4)	69.5 (60.3, 77.7)	<0.001	0.324
Gender, n (%)			0.002	0.183
Female	977 (40.3)	112 (31.5)		
Male	1,449 (59.7)	243 (68.5)		
BMI, kg/m^2^	27.7 (24.2, 31.2)	28.6 (24.8, 32.9)	<0.001	0.199
Ethnicity, n (%)			0.057	0.168
White	1,596 (65.8)	218 (61.4)		
Black	198 (8.2)	24 (6.8)		
Hispanic	60 (2.5)	6 (1.7)		
Asian	55 (2.3)	7 (2.0)		
Other	517 (21.3)	100 (28.2)		
ICU type, n (%)			0.766	0.017
CCU/CVICU	1,456 (60.0)	216 (60.8)		
Other	970 (40.0)	139 (39.2)		
Clinical scores				
SAPS II	38.0 (31.0, 47.0)	41.0 (35.0, 48.0)	<0.001	0.238
SOFA	6.0 (3.0, 9.0)	8.0 (5.0, 10.0)	<0.001	0.507
RASS score	0.0 (−3.0, 0.0)	−4.0 (−5.0, −1.0)	<0.001	0.602
CCI score	8.0 (6.0, 9.0)	7.0 (5.0, 9.0)	<0.001	0.218
Vasoactive-inotropic score	0.0 (0.0, 8.0)	4.1 (0.0, 12.3)	<0.001	0.176
Comorbidities, n (%)				
Congestive heart failure	1,485 (61.2)	183 (51.5)	<0.001	0.196
Chronic pulmonary disease	708 (29.2)	98 (27.6)	0.540	0.035
Diabetes	1,101 (45.4)	145 (40.8)	0.108	0.092
Cerebrovascular disease	411 (16.9)	56 (15.8)	0.583	0.032
Chronic renal disease	891 (36.7)	95 (26.8)	<0.001	0.215
Liver disease	213 (8.8)	35 (9.9)	0.505	0.037
Tumor	229 (9.4)	36 (10.1)	0.674	0.024
Sepsis	1,285 (53.0)	244 (68.7)	<0.001	0.327
Revascularization, n (%)	932 (38.4)	172 (48.5)	<0.001	0.203
Mechanical ventilation, n (%)	1,183 (48.8)	324 (91.3)	<0.001	1.047
RRT, n (%)	306 (12.6)	39 (11.0)	0.385	0.05
Sedative-analgesic medications, n (%)				
Propofol	1,056 (43.5)	314 (88.5)	<0.001	1.077
Midazolam	577 (23.8)	102 (28.7)	0.043	0.113
Fentanyl	1,137 (46.9)	280 (78.9)	<0.001	0.702
medication, n (%)				
Antiplatelet	2,204 (90.8)	309 (87.0)	0.023	0.122
ACEI/ARB	1,200 (49.5)	166 (46.8)	0.341	0.054
Statin	2097 (86.4)	307 (86.5)	0.983	0.001
Beta blockers	1966 (81.0)	298 (83.9)	0.189	0.076
Initial vital signs at ICU admission				
Heart rate, beats/min	82.7 (73.0, 92.7)	83.5 (76.7, 94.3)	0.009	0.144
MBP, mmHg	75.1 (69.3, 82.0)	74.9 (70.5, 79.1)	0.557	0.054
Respiratory rate, beats/min	19.3 (17.2, 21.9)	19.1 (17.1, 21.8)	0.351	0.049
Temperature, °C	36.8 (36.5, 37.0)	36.9 (36.7, 37.2)	<0.001	0.376
SpO_2_, %	97.1 (95.7, 98.4)	97.8 (96.4, 98.8)	<0.001	0.391
Laboratory tests				
WBC, 10^9^/L	11.6 (8.4, 15.9)	12.1 (8.9, 16.9)	0.150	0.032
Hemoglobin g/dL	10.8 (9.0, 12.7)	10.5 (8.9, 12.6)	0.390	0.024
Platelet, 10^9^/L	208.0 (152.0, 274.0)	188.0 (144.0, 255.5)	0.005	0.094
BUN, mg/dL	26.0 (17.0, 45.0)	21.0 (15.0, 31.5)	<0.001	0.273
Creatinine, mg/dL	1.3 (0.9, 2.1)	1.1 (0.9, 1.6)	<0.001	0.107
Calcium level, mg/dL	8.5 (8.0, 9.0)	8.4 (8.0, 8.9)	0.794	0.01
Potassium level, mEq/L	4.3 (3.9, 4.8)	4.4 (4.0, 4.9)	0.125	0.124
Lactate, mmol/L	1.9 (1.5, 2.5)	1.9 (1.4, 2.9)	0.338	0.057
pH	7.4 (7.3, 7.4)	7.4 (7.3, 7.4)	0.707	0.023
pO_2_ level, mmHg	106.0 (67.0, 173.6)	141.0 (58.5, 298.0)	<0.001	0.34
pCO_2_ level, mmHg	40.5 (36.1, 44.9)	41.0 (36.0, 47.0)	0.028	0.125
Glucose, mg/dl	144.0 (114.0, 200.0)	133.0 (110.0, 175.5)	<0.001	0.151

CCU: coronary care unit; CVICU: cardiovascular intensive care unit; SAPS II: simplified acute physiology score II; SOFA: sequential organ failure assessment score; RASS score: Richmond agitation-Sedation scale score; CCI score: Charlson comorbidity index score; RRT: renal replacement therapy; ACEI: angiotensin-converting enzyme inhibitor; ARB: angiotensin receptor blocker; MBP: mean blood pressure; WBC: white blood cell; Revascularization: percutaneous coronary intervention and coronary angioplasty bypass grafting; Vasoactive-inotropic score = (0.1×dopamine dose) + (1×dobutamine dose) + (1×epinephrine dose) + (100×norepinephrine dose) + (100×phenylephrine dose) + (10×vasopressin dose) + (100×milrinone dose).

In the original cohort, patients in the DEX group were characterized by being younger, more likely to be male, having a higher BMI, and exhibiting higher heart rate, temperature and SpO_2_ compared to the non-DEX group. The SAPSII and SOFA scores, used to assess disease severity, were also higher in the DEX group. Moreover, DEX group had lower rates of heart failure and chronic kidney disease, but a higher incidence of sepsis. Variations in sedative and analgesic drug usage were noted between the two groups, with the DEX group showing deeper levels of sedation (higher RASS score). In terms of treatment, a larger proportion of patients in the DEX group underwent revascularization and mechanical ventilation. Notably, there was no statistically significant difference in white blood cell count levels at baseline.

Propensity scores were calculated using predetermined covariates. Within the matched cohort, 349 patients in DEX group were compared with 349 patients in non-DEX group, showing significantly less covariate imbalance between the two groups. The quality of the matched samples was confirmed by graphing the probability density plot of two groups (Supplementary Figure S1) and calculating the standardized mean difference (SMD) (Supplementary Table S1). In the matched cohort, most covariates had SMD values less than 0.1 (Supplementary Figure S2). We included unbalanced covariates in subsequent regression analyses and subgroup analyses to eliminate interference.

### 3.2 Primary outcome

**FIGURE 2:**
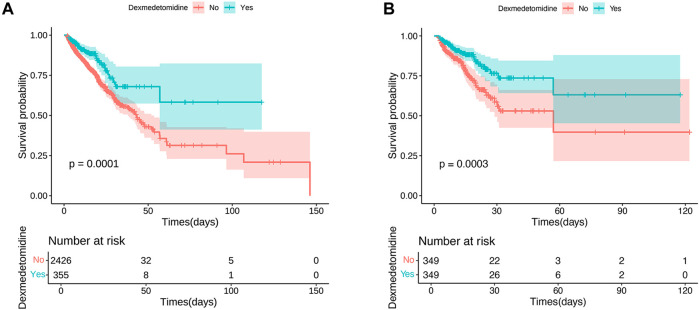
Kaplan–Meier survival curves of in-hospital mortality risk. **(A)** The original population of in-hospital mortality risk; **(B)** After propensity score matching adjustment of in-hospital mortality risk.

The KM survival curve indicated that the in-hospital mortality were lower in the DEX group compared to the non-DEX group, with statistically significant differences (*p* = 0.0001; Figure 2A). These results remained consistent after PSM processing (*p* = 0.0003; Figure 2B).

None of the Variance Inflation Factors (VIFs) exceed 5 (Supplementary Table S2). This suggests that there is no multicollinearity present among the variables. The initial univariate Cox regression analysis indicated a significant 45% decrease in the risk of in-hospital death associated with DEX use in the original cohort, with a HR of 0.55 (95% CI 0.40–0.74, *p* = 0.0001). Following comprehensive adjustments for various confounders in a multivariate Cox regression analysis, DEX use was still found to be significantly linked to a 50% reduction in the risk of in-hospital death, with a HR of 0.50 (95% CI 0.35–0.71, *p* < 0.0001). After PSM, the crude models demonstrated that DEX use was related to a decreased mortality risk with a HR of 0.50 (95% CI: 0.34–0.74, *p* = 0.0004). The PSM models also showed similar results with a HR of 0.49 (95% CI: 0.31–0.77, *p* = 0.0022) in the fully adjusted models (Table 2).

**TABLE 2:** Cox proportional hazards models for in-hospital all-cause mortality in AMI patients treated with early DEX.

Models	Original cohort		PSM cohort	
	HR (95% CI)	*p*-value	HR (95% CI)	*p*-value
Crude model	0.55 (0.40, 0.74)	0.0001	0.50 (0.34, 0.74)	0.0004
Model 1	0.57 (0.42, 0.79)	0.0005	0.49 (0.33, 0.72)	0.0003
Model 2	0.57 (0.41, 0.77)	0.0004	0.50 (0.34, 0.74)	0.0005
Model 3	0.37 (0.26, 0.53)	<0.0001	0.50 (0.33, 0.76)	0.0012
Model 4	0.42 (0.29, 0.60)	<0.0001	0.48 (0.31, 0.74)	0.0008
Model 5	0.45 (0.32, 0.65)	<0.0001	0.48 (0.31, 0.74)	0.0010
Model 6	0.50 (0.35, 0.71)	<0.0001	0.49 (0.31, 0.77)	0.0022

Model one was adjusted for demographic features, including age, gender, BMI, ethnicity and ICU type. Model two was additionally adjusted for comorbidities, including congestive heart failure, cerebrovascular disease, chronic pulmonary disease, chronic renal disease, liver disease, tumor and sepsis. Model three was additionally adjusted for clinical scores, including SAPS II, SOFA, RASS score, CCI score and vasoactive-inotropic score. Model four was additionally adjusted for vital signs, including heart rate, respiratory rate, MBP, temperature, and SpO2. Model five was additionally adjusted for laboratory tests, including WBC, BUN, calcium, creatinine, glucose, pH, pO2, pCO2 and lactate. Model six was additionally adjusted for clinical therapy, including revascularization, mechanical ventilation, RRT and medication.

The COX regression model was assessed using the Schoenfeld residual method (Supplementary Table S3). The results (global test: *p* = 0.4984) confirmed that the Cox regression model meets the proportional hazards (PH) assumption, suggesting that the HR estimation is reliable.

We further analyzed the dose-response relationship. Comparing the survival differences between different average dose rates and cumulative dose (over a 7-day period), we discovered that in-hospital mortality decreased as the dose of DEX increased compared to the non-DEX group (Supplementary Figure S3).

### 3.3 Secondary outcomes and adverse events

In the analysis of secondary outcomes (Table 3), we observed a decrease in the risk of myocardial infarction at 30 days (Original cohort: HR: 0.63, 95%CI: 0.46–0.84; Matched cohort: HR: 0.59, 95%CI: 0.40–0.88) and 90-day mortality (Original cohort: HR: 0.71, 95%CI: 0.55–0.92; Matched cohort: HR: 0.70, 95%CI: 0.50–0.97) in both the original and matched cohorts when adjusted in the COX model.

**TABLE 3:** Secondary outcomes and adverse events associated with early DEX use in AMI patients.

	Original cohort				PSM cohort			
	DEX (n = 355)	Non-DEX (n = 2,426)	Effect size (95% CI)	*p*-value	DEX (n = 349)	Non-DEX (n = 349)	Effect size (95% CI)	*p*-value
secondary outcomes								
30-day mortality	60 (16.9%)	522 (21.5%)	0.63 (0.46, 0.84)	0.0020	56 (16.1%)	87 (24.9%)	0.59 (0.40, 0.88)	0.0100
90-day mortality	82 (23.1%)	673 (27.7%)	0.71 (0.55, 0.92)	0.0010	78 (22.4%)	102 (29.2%)	0.70 (0.50, 0.97)	0.0401
Length of ICU stay, day	4.20 (2.95–6.76)	3.66 (2.66–5.62)	0.71 (0.14, 1.28)	0.0142	4.16 (2.91, 8.07)	4.17 (2.93, 6.68)	0.52 (−0.43, 1.47)	0.2817
Length of hospital stay, day	11.23 (7.53–18.40)	8.96 (5.82–14.18)	1.48 (0.22, 2.75	0.0217	11.21 (7.52–17.90	10.90 (7.09–16.23	1.02 (−0.80, 2.84	0.2719
AKI within 7d	294 (83.7%)	1944 (80.1%)	0.98 (0.69, 1.41	0.9242	291 (83.4%)	290 (83.1%)	0.85 (0.51, 1.41	0.5199
Adverse events								
Bradycardia	51 (14.4%)	393 (16.2%)	0.75 (0.51, 1.10)	0.1369	49 (14.0%)	66 (18.9%)	0.72 (0.43, 1.22)	0.2217
Hypotension	170 (47.9%)	1,201 (49.5%)	0.94 (0.75, 1.17)	0.5690	167 (47.9%)	190 (54.4%)	0.71 (0.49, 1.02)	0.0740

Bradycardia: Pulse rate less than 50 beats/min; Hypotension: Systolic blood pressure less than 80 mmHg.

In the original cohort, it was noted that the use of DEX was linked to extended stays in the ICU (3.66 days vs. 4.20 days; β: 0.71; *p* = 0.0142) and longer hospital stays (8.96 days vs. 11.23 days; β: 1.48; *p* = 0.0217). However, this correlation ceased to exist after PSM.

In both cohorts, we found no evidence that the use of DEX reduced the risk of AKI within 7 days of hospitalization in patients with MI. (original cohort: 83.7% vs. 80.1%; OR 0.98; *p* = 0.9242; Matched cohort: 83.4% vs. 83.1%; OR 0.85; *p* = 0.5199).

Regarding adverse events, the incidence of hypotension in the DEX group compared to the non-DEX group showed similar rates in both the original cohort (47.9% vs. 49.5%; OR 0.94; *p* = 0.5690) and the matched cohort (47.9% vs. 54.4%; OR 0.71; *p* = 0.0740). Similarly, the occurrence of bradycardia also exhibited no significant difference between the two groups in the original cohort (14.4% vs. 16.2%; OR 0.75; *p* = 0.1369) and the matched cohort (14.0% vs. 18.9%; OR 0.72; *p* = 0.2217).

### 3.4 Causal mediation analysis

In the matched cohort, we observed a higher decrease in white blood cell count from day 1 to day 4 in the DEX group compared to the no-dexmedetomidine group (−3.60 vs. −3.19 × 109/L; *p* < 0.001).

**FIGURE 3:**
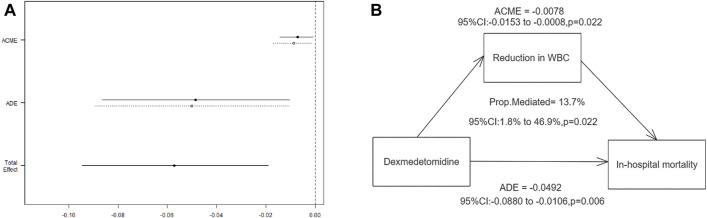
Mediation by WBC reduction of the association between dexmedetomidine and in-hospital mortality. **(A)** Effect estimates and confidence intervals for ACME, ADE, and total effect; **(B)** Mediation pathway diagram showing the role of WBC reduction. ACME: average causal mediation effect; ADE: average direct effect.

CMA analysis (Figures 3A, B) revealed a significant correlation between early DEX treatment and in-hospital mortality, with a direct effect of −0.05 (95% CI, −0.09, −0.01; *p* = 0.006). Furthermore, the study found that 13.7% (95% CI, 1.8%–46.9%, *p* = 0.022) of the beneficial effect of DEX medication on reducing in-hospital mortality in MI patients was mediated by the reduction in WBC.

### 3.5 Subgroup analysis

**FIGURE 4:**
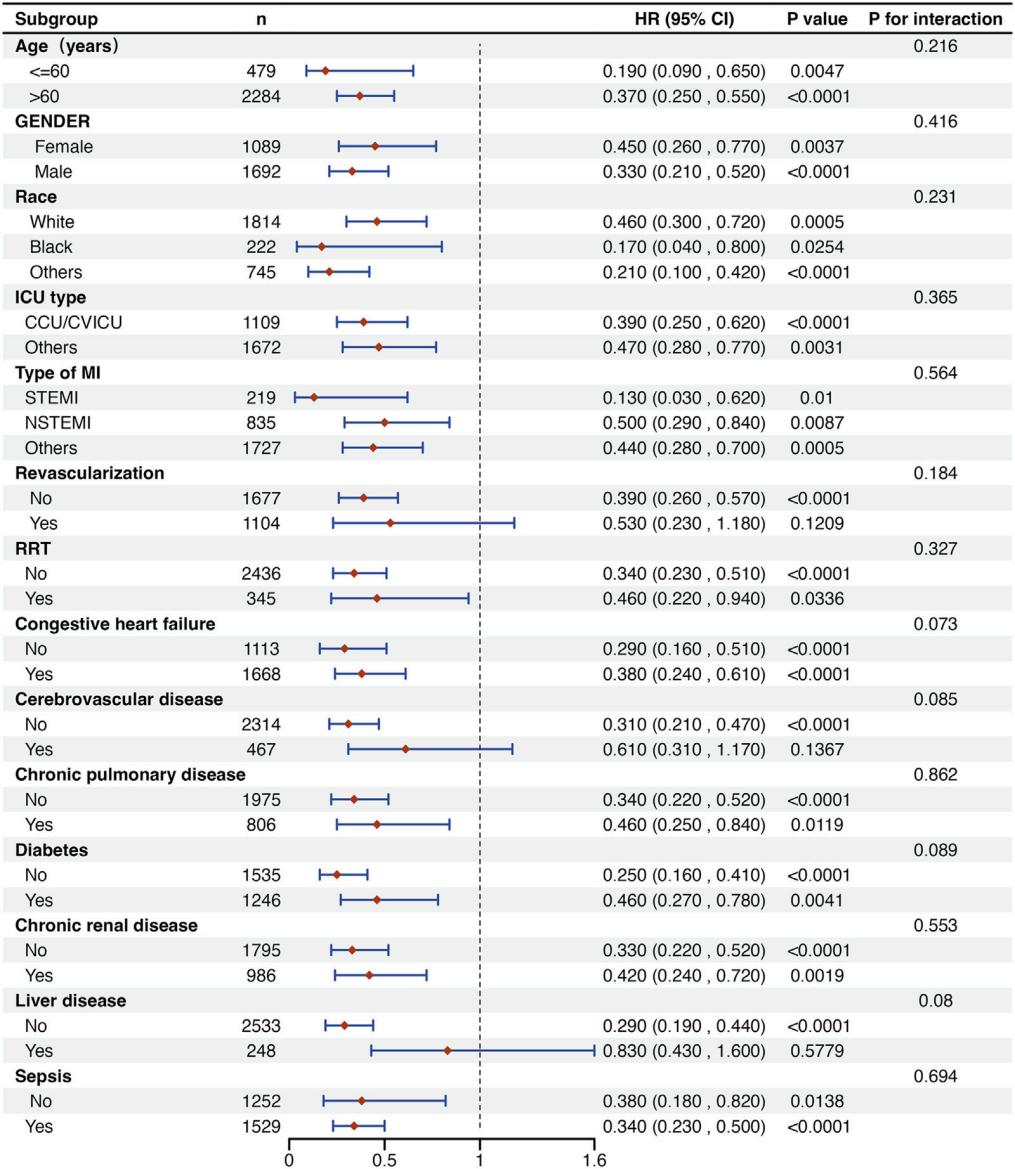
Subgroup analysis of the association between DEX use and outcomes in critically ill patients with MI. CCU: coronary care unit; CVICU: cardiovascular intensive care unit; STEMI: ST-segment elevation myocardial infarction; NSTEMI: Non-ST-segment elevation myocardial infarction; RRT: renal replacement therapy.

In addition, a detailed subgroup analysis was conducted on in-hospital mortality (Figure 4). The findings indicated that the use of DEX was associated with increased survival rates among patients with MI across most subgroups. However, there was no significant improvement in survival outcomes when examining patients with comorbid cerebrovascular disease and liver disease, as well as those who underwent revascularization. Additionally, no significant interactions were found between the DEX group and the non-DEX group across all strata.

## 4 Discussion

In this study, we discovered that DEX reduces in-hospital mortality, 30-day mortality and 90-day mortality in critically ill patients with AMI, and a potential dose-dependent relationship was observed between DEX administration and in-hospital mortality. Subsequently, we used CMA to delve into its underlying mechanism and observed that DEX’s positive impact on the survival outcomes of myocardial infarction patients is linked, at least partially, to its anti-inflammatory properties. Furthermore, our findings indicated that its administration may have led to prolonged ICU and hospital stays. Lastly, with regard to safety concerns, our study did not identify an increased risk of hypotension or bradycardia associated with DEX use during hospitalization.

Our study is the first to demonstrate that DEX can enhance survival outcomes in patients with AMI. Previous clinical research has indicated that DEX has the potential to shield the ischemic heart from IRI during cardiac surgery (Ríha et al., 2012; Chen et al., 2015; Chi et al., 2016; Elgebaly et al., 2020). A comprehensive meta-analysis involving 48 trials and 6,273 participants revealed that the perioperative administration of DEX during cardiac surgery led to a decrease in short-term mortality (Poon et al., 2023). A limited number of studies have explored the impact of DEX on the prognosis of individuals with AMI. Jiang Xiaowei et al. conducted a study comparing the effects of midazolam, propofol, and DEX on the prognoses of critically ill patients with AMI (Jiang and Yan, 2021); however, their study included only 28 patients in the DEX group, leading to a small sample size that prevented the detection of statistically significant positive outcomes of DEX on the survival of myocardial infarction patients. Our study, with a larger sample size, affirmed the beneficial effects of this drug in individuals with AMI, and the results remained robust even among individuals who did not undergo revascularization or among those with different types of myocardial infarction.

Our study also suggests that DEX’s improved prognosis in myocardial infarction patients may be linked to its anti-inflammatory properties. The leukocyte response seen during AMI has traditionally been viewed as a manifestation of acute phase inflammation. This response is triggered by necrotic injury and is seen as a crucial part of the healing process; however, inflammation can be a double-edged sword. While it helps in repairing tissue damage, an excessive inflammatory response can also contribute to myocardial cell damage, resulting in a poorer prognosis for myocardial infarction patients (Bodi et al., 2008; Öcal et al., 2022). Therefore, the failure of certain anti-inflammatory treatment strategies (such as clinical trials of methylprednisolone in myocardial infarction treatment) (Roberts et al., 1976) may be attributed to the indiscriminate inhibition of the inflammatory process, which disrupts crucial early inflammatory signals. Although this may reduce early inflammatory damage, it may also delay healing and collagen deposition.

Some studies have found that DEX can activate signaling pathways through G proteins such as PI3K/Akt and MEK1-2-ERK1/2, reducing the inflammatory responses and apoptosis caused by ischemia-reperfusion, thus decreasing myocardial infarct size (Ibacache et al., 2012; Sulaiman et al., 2012). This indicates that DEX may serve as a targeted anti-inflammatory drug with a more rational pathway, effectively inhibiting inflammation without disrupting the repair response. Our study found a notable difference in the decreased white blood cell counts between the DEX group and non-DEX groups and confirmed, through CMA, that DEX’s effect on the survival of myocardial infarction patients was proportionally mediated by a reduction in white blood cell counts. These findings suggest that DEX may alleviate the inflammatory response in myocardial infarction patients, leading to improved prognoses.

In addition, it should be noted that reducing the inflammatory response may be just one of the ways in which DEX protects the ischemic heart. It may also provide protection by improving microcirculatory dysfunction (Lawrence et al., 1996), reducing oxidative stress (Han et al., 2019), and through anti-arrhythmic mechanisms (Hammer et al., 2008).

We also found a potential association between DEX and extended ICU and hospital stays in patients with myocardial infarction; however, previous studies have yielded conflicting results (Tan and Ho, 2010; Patanwala and Erstad, 2016; Hu et al., 2022), possibly due to variations in patient populations and discharge criteria across different healthcare facilities. The extended hospital stays observed in this study could be attributed to lower mortality rates in the DEX group, leading to longer treatment and recovery periods. Furthermore, prolonged hospitalization is correlated with a higher risk of delirium (McNicoll et al., 2003), leading to a greater likelihood of DEX use for sedation and as an anti-delirium therapy in patients with longer hospital stays.

Our study did not determine whether early DEX use can reduce AKI risk within 7 days of hospitalization. Patients with myocardial infarction are at risk for AKI, especially those who underwent percutaneous coronary intervention and received contrast agent injection (Dodson et al., 2019). Therefore, elderly patients or those with a history of chronic kidney disease should be vigilant about the possibility of AKI following a myocardial infarction. A study based on the MIMIC-IV database found a positive impact on renal function improvement in sepsis patients (Hu et al., 2022). Future research should focus on designing more rigorous clinical studies to investigate the renal protective effects of dexmedetomidine in myocardial infarction patients.

In terms of safety outcomes, our findings did not indicate that DEX raised the risk of hypotension or bradycardia, aligning with the results of numerous extensive clinical investigations (Tan and Ho, 2010; Song et al., 2023). Nevertheless, a handful of case reports have hinted at potentially fatal complications associated with DEX use, including pulseless electrical activity (Gerlach and Murphy, 2009) and refractory cardiogenic shock (Hutchens and Thorborg, 2009) in specific populations. These observations underscore the importance of thoroughly evaluating the potential contraindications of DEX and exercising caution when using this medication.

## 5 Limitations

This study has several limitations. First, it was a retrospective study that used the MIMIC-IV database and, despite rigorous propensity score matching and multivariate analyses, there is a possibility of unmeasured confounding factors influencing the outcomes. Second, the administration practices of DEX were not uniform. The relationship between the timing of its administration and the occurrence of myocardial infarction is unclear. This distinction is crucial when treating patients with myocardial infarction, as in many cases, drug treatments can only be administered after the onset of the disease. Third, due to the absence of laboratory indicators in the MIMIC-IV database, we were unable to extract more representative inflammatory markers, such as TNF-α, IL-6, and CRP, for validation. Finally, using multiple imputations to fill in missing data can lead to deviations from the true values. To mitigate the potential for bias introduced by imputed data, sensitivity analyses were conducted to evaluate the robustness of the imputation results in studies using multiple imputation. Therefore, further well-designed, multicenter clinical trials are necessary in the future to investigate the prognostic association between DEX and myocardial infarction.

## 6 Conclusion

Dexmedetomidine treatment is linked to a lower risk of mortality in individuals with acute myocardial infarction, partly due to its anti-inflammatory properties. Overall, we posit that dexmedetomidine could be a valuable option in managing myocardial infarction clinically; nevertheless, further multicenter, large-scale, and meticulously planned randomized controlled trials are imperative to substantiate this conclusion.

## Data Availability

Publicly available datasets were analyzed in this study. This data can be found here: https://mimic-iv.mit.edu/.
