# Supplementary material for: Dexmedetomidine administration is associated with improved outcomes in critically ill patients with acute myocardial infarction partly through its anti-inflammatory activity
Source: Front Pharmacol. 2024 Aug 22;15:1428210. doi: 10.3389/fphar.2024.1428210 (PMC11375293; doi:10.3389/fphar.2024.1428210)
Supplement: Supplementary file 1 [file DataSheet1.docx]

Supplementary Material

**Supplementary Fig S1**. Probability density plot of DEX and non-DEX group before and after propensity score matching.

*
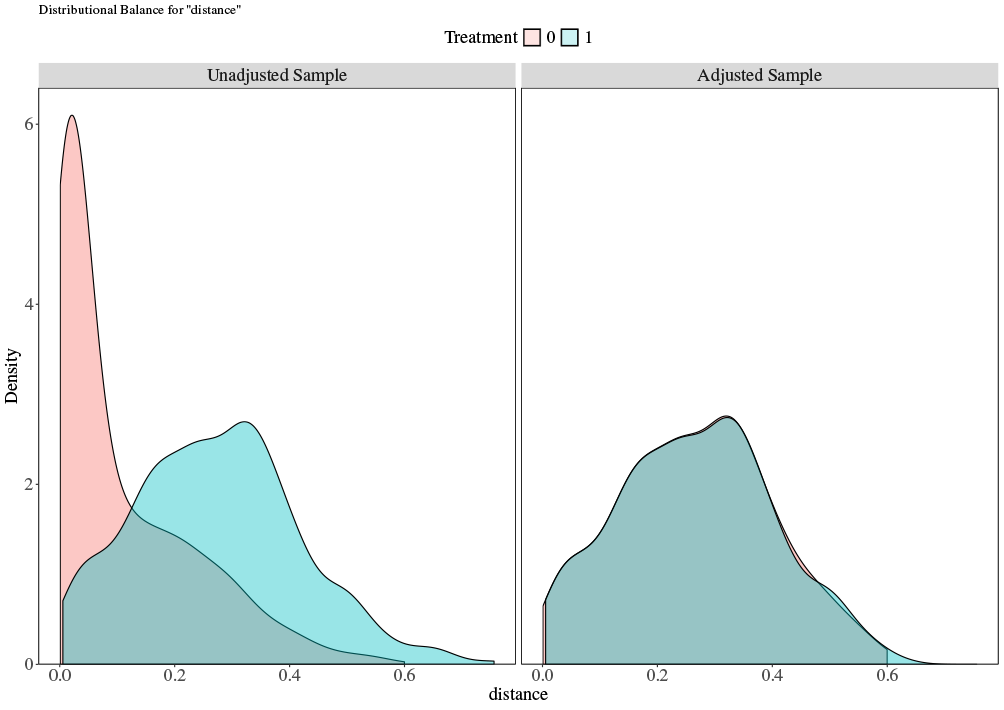
*

**Supplementary** Fig S2. Comparison chart displaying the standardized mean difference before and after propensity score matching.

*
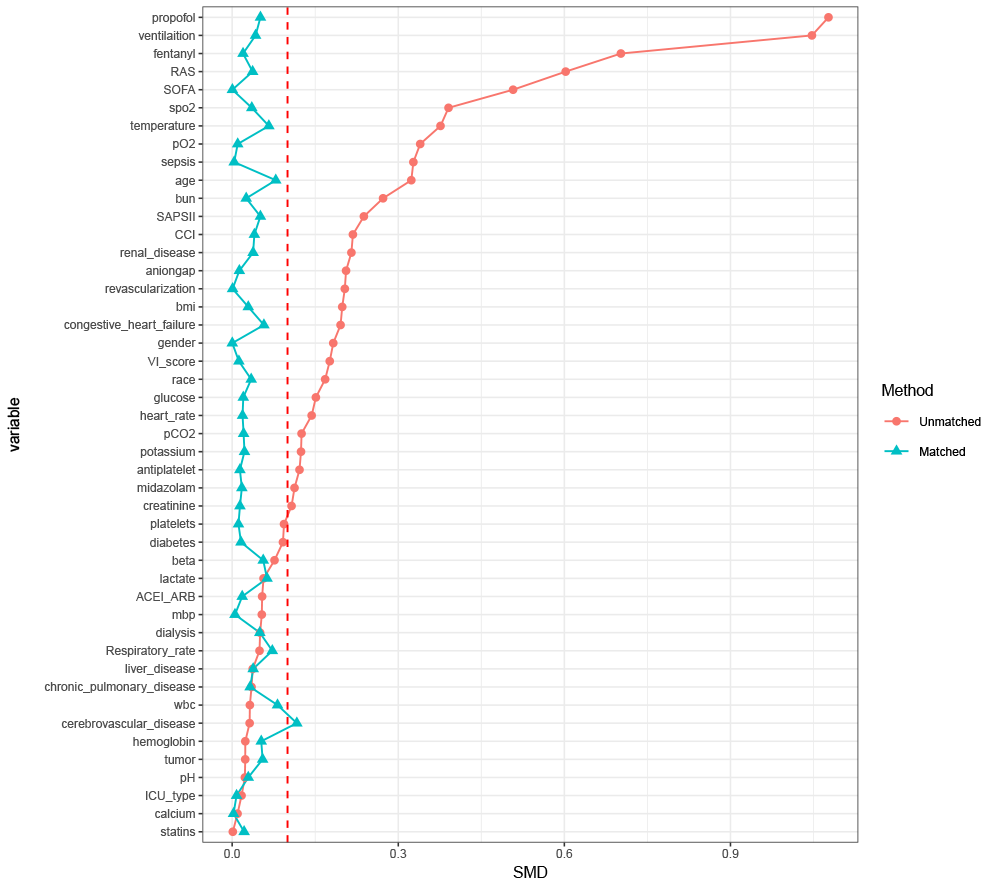
*

**Supplementary** Fig S3.(A) K-M survival curves among groups with different average dose rates.

*
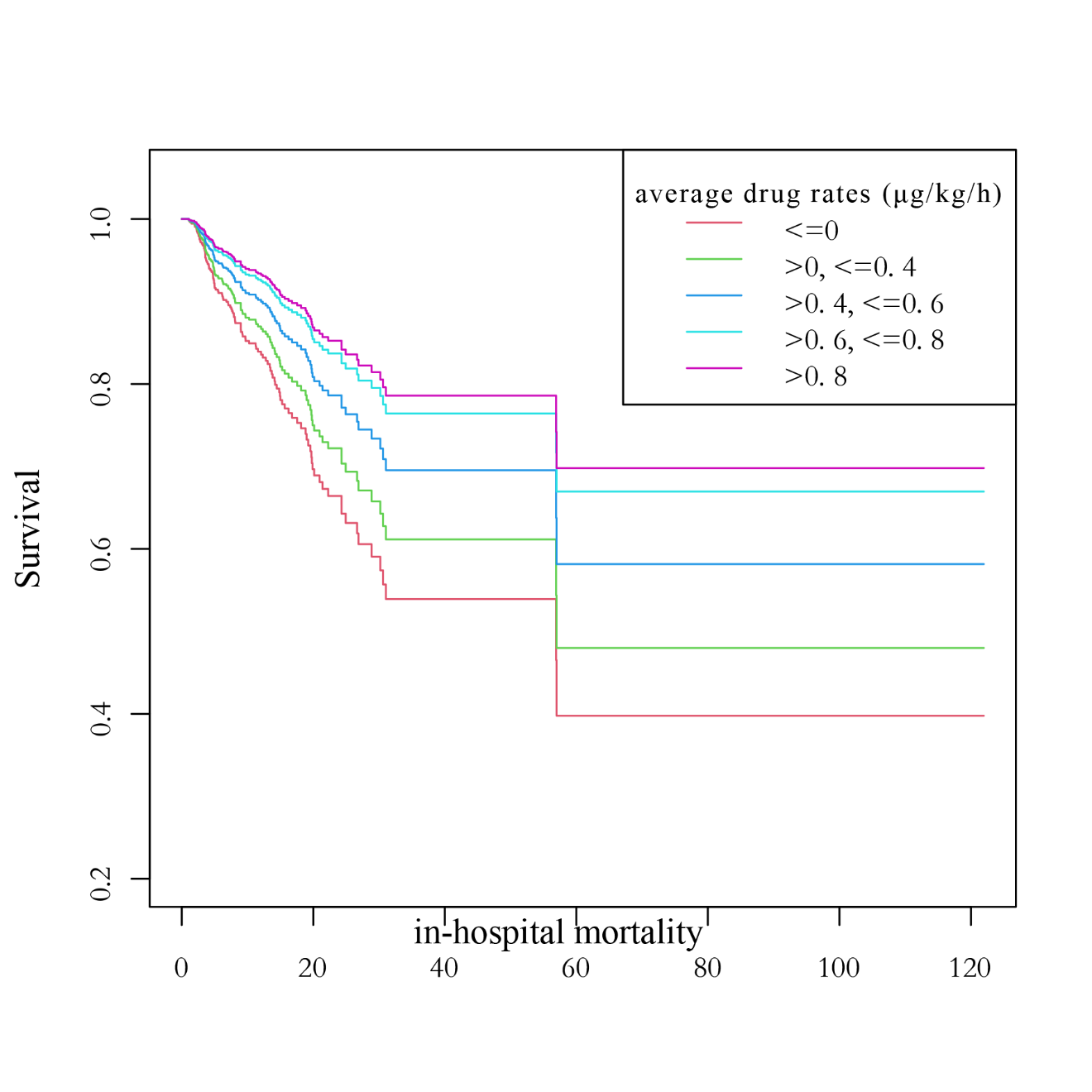
*

**Supplementary** Fig S3.(B) K-M survival curves among groups with different cumulative dose over a 7-day period.


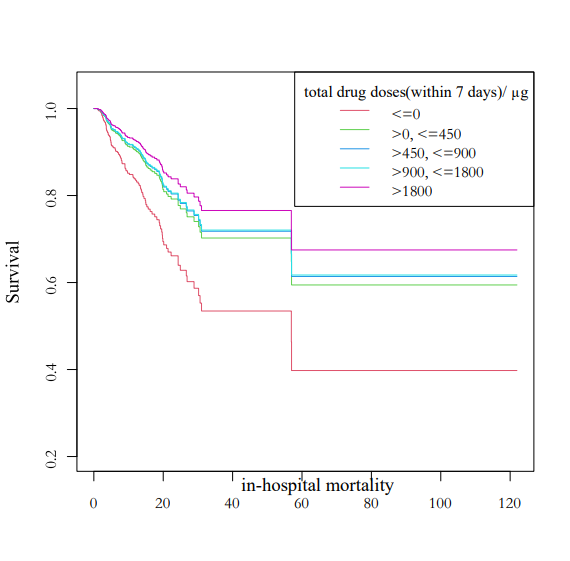


**Supplementary Tab S1.** Baseline characteristics between two groups after PSM.

| After PSM | | | | |
| --- | --- | --- | --- | --- |
| Characteristics | Non-DEX group | DEX group | *p* value | SMD |
| N | (n=349) | (n=349) |  |  |
| Age | 69.6 (60.5, 77.6) | 69.6 (60.3, 77.7) | 0.997 | 0.079 |
| Gender, n (%) |  |  | 0.871 | <0.001 |
| Female | 109 (31.2) | 111 (31.8) |  |  |
| Male | 240 (68.8) | 238 (68.2) |  |  |
| BMI,kg/m^2^ | 28.2 (24.7, 32.5) | 28.7 (24.8, 32.8) | 0.647 | 0.029 |
| Ethnicity, n (%) |  |  | 0.893 | 0.034 |
| White | 217 (62.2) | 217 (62.2) |  |  |
| Black | 23 (6.6) | 23 (6.6) |  |  |
| Hispanic | 8 (2.3) | 6 (1.7) |  |  |
| Asian | 4 (1.2) | 7 (2.0) |  |  |
| Other | 97 (27.8) | 96 (27.5) |  |  |
| ICU type, n (%) |  |  | 0.938 | 0.008 |
| CCU/CVICU | 134 (38.4) | 133 (38.1) |  |  |
| Other | 215 (61.6) | 216 (61.9) |  |  |
| Clinical scores | | | | |
| SAPS II | 42.0 (34.0, 52.0) | 40.0 (35.0, 48.0) | 0.252 | 0.051 |
| SOFA | 8.0 (5.0, 11.0) | 8.0 (5.0, 10.0) | 0.715 | <0.001 |
| RASS score | -3.0 (-5.0, 0.0) | -4.0 (-5.0, -1.0) | 0.386 | 0.037 |
| CCI score | 7.0 (5.0, 9.0) | 7.0 (5.0, 9.0) | 0.815 | 0.040 |
| Vasoactive-inotropic score | 4.8 (0.0, 12.7) | 3.7 (0.0, 11.8) | 0.320 | 0.012 |
| Comorbidities, n (%) |  |  |  |  |
| Congestive heart failure | 188 (53.9) | 182 (52.2) | 0.649 | 0.057 |
| Chronic pulmonary disease | 99 (28.4) | 98 (28.1) | 0.933 | 0.032 |
| Diabetes | 142 (40.7) | 142 (40.7) | 1.000 | <0.001 |
| Cerebrovascular disease | 78 (22.4) | 55 (15.8) | 0.027 | 0.117 |
| Chronic renal disease | 96 (27.5) | 94 (26.9) | 0.865 | 0.038 |
| Liver disease | 32 (9.2) | 33 (9.5) | 0.896 | 0.038 |
| Tumor | 27 (7.7) | 35 (10.0) | 0.287 | 0.055 |
| Sepsis | 219 (65.5) | 238 (68.2) | 0.678 | 0.004 |
| Revascularization, n (%) | 169 (48.4) | 172 (49.3) | 0.820 | 0.001 |
| Mechanical ventilation, n (%) | 317 (90.8) | 318 (91.1) | 0.895 | 0.042 |
| RRT, n (%) | 52 (14.9) | 36 (10.3) | 0.068 | 0.050 |
| Sedative-analgesic medications, n (%) | | | | |
| Propofol | 300 (86.0) | 308 (88.3) | 0.366 | 0.051 |
| Midazolam | 97 (27.8) | 100 (28.7) | 0.801 | 0.017 |
| Fentanyl | 279 (79.9) | 274 (78.5) | 0.641 | 0.020 |
| medication, n (%) | | | | |
| Antiplatelet | 296 (84.8) | 307 (88.0) | 0.225 | 0.014 |
| ACEI/ARB | 157 (45.0) | 165 (47.3) | 0.544 | 0.018 |
| Statin | 283 (81.1) | 305 (87.4) | 0.821 | 0.022 |
| Beta blockers | 281 (80.5) | 295 (84.5) | 0.163 | 0.056 |
| Initial vital signs at ICU admission | | | | |
| Heart rate, beats/min | 84.5 (77.0, 93.8) | 83.2 (76.5, 93.7) | 0.736 | 0.019 |
| MBP, mmHg | 74.6 (69.5, 80.0) | 75.0 (70.6, 79.3) | 0.447 | 0.005 |
| Respiratory rate, beats/min | 18.9 (17.0, 21.5) | 19.0 (17.1, 21.7) | 0.837 | 0.073 |
| Temperature, °C | 36.9 (36.7, 37.3) | 36.9 (36.7, 37.2) | 0.395 | 0.066 |
| SpO_2_, % | 97.9 (96.5, 99.0) | 97.8 (96.4, 98.9) | 0.795 | 0.035 |
| Laboratory tests | | | | |
| WBC, 10^9^ /L | 12.5 (8.6, 17.3) | 12.1 (9.0, 16.6) | 0.801 | 0.082 |
| Hemoglobin, g/dL | 11.0 (8.8, 12.8) | 10.5 (8.9, 12.5) | 0.325 | 0.053 |
| Platelet, 10^9^ /L | 197.0 (145.0, 266.0) | 187.0 (144.0, 256.0) | 0.426 | 0.011 |
| BUN, mg/dL | 23.0 (16.0, 37.0) | 21.0 (15.0, 32.0) | 0.077 | 0.025 |
| Creatinine, mg/dL | 1.10(0.9, 1.8) | 1.1 (0.9, 1.6) | 0.058 | 0.014 |
| Calcium level, mg/dL | 8.3 (8.0, 9.0) | 8.4 (8.0, 8.9) | 0.285 | 0.002 |
| Potassium level, mEq/L | 4.4 (4.0, 4.9) | 4.4 (4.0, 4.8) | 0.180 | 0.022 |
| Lactate, mmol/L | 2.0 (1.4, 2.6) | 1.9 (1.4, 2.8) | 0.852 | 0.063 |
| pH | 7.4 (7.3, 7.4) | 7.4 (7.3, 7.4) | 0.680 | 0.029 |
| pO_2_ level, mmHg | 131.0 (67.0, 295.0) | 150.0 (61.0, 301.0) | 0.924 | 0.010 |
| pCO_2_ level, mmHg | 41.3 (37.0, 47.0) | 41.0 (36.0, 47.0) | 0.605 | 0.021 |
| Glucose, mg/dl | 137.0 (112.0, 183.0) | 132.0 (110.0, 176.0) | 0.685 | 0.020 |

CCU: coronary care unit; CVICU: cardiovascular intensive care unit; SAPS II: simplified acute physiology score II; SOFA: sequential organ failure assessment score; RASS score: Richmond agitation-Sedation scale score; CCI score: Charlson comorbidity index score; RRT: renal replacement therapy; ACEI: angiotensin-converting enzyme inhibitor; ARB: angiotensin receptor blocker; MBP: mean blood pressure; WBC: white blood cell; Revascularization: percutaneous coronary intervention and coronary angioplasty bypass grafting; Vasoactive-inotropic score = (0.1×dopamine dose) + (1×dobutamine dose) + (1×epinephrine dose) + (100×norepinephrine dose) + (100×phenylephrine dose) + (10×vasopressin dose) + (100×milrinone dose).

**Supplementary Tab S2.** Variance inflation factors (VIFs) of the variables

| Variable | VIF | 1/VIF |
| --- | --- | --- |
| Age | 2.32 | 0.430838 |
| Gender | 1.18 | 0.850058 |
| BMI | 1.24 | 0.803415 |
| Ethnicity | 1.10 | 0.908714 |
| ICU type | 2.36 | 0.424579 |
| SAPS II | 2.27 | 0.439824 |
| SOFA | 2.51 | 0.398873 |
| RASS score | 1.47 | 0.681094 |
| CCI score | 4.05 | 0.246914 |
| Vasoactive-inotropic score | 1.42 | 0.701968 |
| Congestive heart failure | 1.51 | 0.661230 |
| Chronic pulmonary disease | 1.43 | 0.700938 |
| Diabetes | 1.78 | 0.562363 |
| Cerebrovascular disease | 1.38 | 0.722278 |
| Chronic renal disease | 1.43 | 0.700938 |
| Liver disease | 1.37 | 0.732507 |
| Tumor | 2.24 | 0.446617 |
| Sepsis | 1.19 | 0.838493 |
| Revascularization | 2.59 | 0.385878 |
| Mechanical ventilation | 1.46 | 0.683418 |
| RRT | 1.65 | 0.605305 |
| Propofol | 1.47 | 0.680810 |
| Midazolam | 1.43 | 0.699162 |
| Fentanyl | 1.36 | 0.737692 |
| Antiplatelet | 1.67 | 0.599932 |
| ACEI/ARB | 1.25 | 0.796900 |
| Statin | 1.71 | 0.583296 |
| Beta blockers | 1.40 | 0.715810 |
| Heart rate | 1.34 | 0.748472 |
| MBP | 1.41 | 0.710678 |
| Respiratory rate | 1.48 | 0.675148 |
| Temperature | 1.25 | 0.803099 |
| SpO2 | 1.41 | 0.708129 |
| WBC | 1.26 | 0.796245 |
| Hemoglobin | 1.60 | 0.623123 |
| Platelet | 1.33 | 0.754515 |
| BUN | 2.46 | 0.406887 |
| Creatinine | 2.19 | 0.455850 |
| Calcium | 1.42 | 0.703401 |
| Potassium | 1.48 | 0.677463 |
| Lactate | 1.69 | 0.591665 |
| pH | 1.20 | 0.830178 |
| pO2 | 2.18 | 0.458703 |
| pCO2 | 1.48 | 0.674083 |
| Glucose | 1.67 | 0.599142 |

**Supplementary Tab S3.** Table of proportional hazards assumption assessment using Schoenfeld Residual Method for Cox model.

|  | rho | chi2 | df | *p* value |
| --- | --- | --- | --- | --- |
| dex_use | -0.00027 | 0.00 | 1 | 0.9948 |
| age | -0.00434 | 0.01 | 1 | 0.9144 |
| bmi | -0.00476 | 0.01 | 1 | 0.9088 |
| heart_rate | 0.01601 | 0.14 | 1 | 0.7034 |
| mbp | -0.06904 | 2.88 | 1 | 0.0897 |
| resp_rate | 0.02570 | 0.36 | 1 | 0.5489 |
| temperatur | 0.00987 | 0.06 | 1 | 0.8142 |
| spo2 | 0.04504 | 1.35 | 1 | 0.2457 |
| ph | 0.02366 | 0.29 | 1 | 0.5934 |
| po2 | 0.04736 | 1.44 | 1 | 0.2306 |
| pco2 | 0.04970 | 1.42 | 1 | 0.2330 |
| lactate | 0.01040 | 0.05 | 1 | 0.8149 |
| hemoglobin | 0.01795 | 0.17 | 1 | 0.6809 |
| platelets | -0.02805 | 0.37 | 1 | 0.5440 |
| wbc | -0.01463 | 0.08 | 1 | 0.7745 |
| aniongap | 0.02775 | 0.39 | 1 | 0.5322 |
| bicarbonate | -0.01608 | 0.13 | 1 | 0.7170 |
| BUN | 0.01966 | 0.22 | 1 | 0.6415 |
| calcium | -0.03275 | 0.66 | 1 | 0.4179 |
| chloride | 0.01688 | 0.14 | 1 | 0.7102 |
| creatinine | -0.06254 | 1.91 | 1 | 0.1664 |
| glucose | -0.04959 | 1.22 | 1 | 0.2702 |
| sodium | 0.00978 | 0.05 | 1 | 0.8295 |
| potassium | 0.00114 | 0.00 | 1 | 0.9785 |
| ras | 0.11515 | 7.00 | 1 | 0.0081 |
| gender | 0.05583 | 1.68 | 1 | 0.1948 |
| race | -0.01084 | 0.07 | 1 | 0.7988 |
| icu_type | -0.02328 | 0.27 | 1 | 0.6008 |
| CCI | 0.03311 | 0.71 | 1 | 0.3985 |
| Congestive heart failure | 0.00863 | 0.04 | 1 | 0.8400 |
| Cerebrovascular disease | -0.00986 | 0.05 | 1 | 0.8154 |
| Chronic pulmonary disease | -0.03438 | 0.65 | 1 | 0.4192 |
| diabetes | -0.04901 | 1.40 | 1 | 0.2371 |
| Chronic renal disease | -0.05664 | 1.84 | 1 | 0.1750 |
| Liver disease | 0.02592 | 0.36 | 1 | 0.5502 |
| tumor | 0.01174 | 0.07 | 1 | 0.7898 |
| sapsii | -0.01131 | 0.07 | 1 | 0.7948 |
| sofa | -0.04538 | 1.26 | 1 | 0.2619 |
| ventilaition | -0.03338 | 0.59 | 1 | 0.4442 |
| RRT | 0.04933 | 1.31 | 1 | 0.2531 |
| Revascularization | -0.00808 | 0.04 | 1 | 0.8473 |
| antiplatelet | 0.02098 | 0.22 | 1 | 0.6379 |
| acei/arb | 0.05773 | 1.72 | 1 | 0.1891 |
| statins | 0.04797 | 1.20 | 1 | 0.2736 |
| beta | 0.04497 | 1.10 | 1 | 0.2946 |
| propofol | 0.04651 | 1.25 | 1 | 0.2629 |
| midazolam | 0.06958 | 2.58 | 1 | 0.1080 |
| fentanyl | 0.01768 | 0.17 | 1 | 0.6817 |
| vi_score | -0.04072 | 0.83 | 1 | 0.3624 |
| sepsis | 0.01623 | 0.15 | 1 | 0.7023 |
|  | | | | |
| Global test |  | 49.37 | 50 | 0.4984 |
